# Ethical considerations of digital health technology in older adult care

**DOI:** 10.1016/S2666-7568(23)00236-2

**Published:** 2024-01

**Authors:** M G Finco, Nabiel Mir, Gillian Gresham, Megan Huisingh-Scheetz

**Affiliations:** Department of Surgery, Baylor College of Medicine, Houston, TX 77030, USA; Department of Medicine, Section of Hematology/Oncology, University of Chicago, Chicago, IL, USA; Department of Medicine, Cedars-Sinai Medical Center, Los Angeles, CA, USA; Department of Medicine, Section of Geriatrics and Palliative Medicine, University of Chicago, Chicago, IL, USA

Digital health technology has the potential to revolutionise geriatric care. The digital divide has decreased among older adults,^1^ and over a third of adults aged 50 years and older in the USA already use technology for health or independence.^2^ Device types are broad (eg, wearable and non-wearable sensors, tablets, telephones, computers, cameras, robots, and voice-activated technology) and have the potential to improve two key health-care domains: monitoring (eg, activity, sleep, glucose, blood pressure, heart rate, falls, frailty, cognitive function, and medication or treatment adherence)^3–7^ and service delivery (eg, remote provider visits, education, reminders, and health information sharing). These technologies could enhance quality of life, improve health care access, enable earlier detection of health issues, and foster patient and caregiver engagement.

Although these technologies have the potential to improve care for older adults, important ethical considerations must be addressed to reduce to the digital divide and health disparities. In this Comment, we outline three crucial ethical considerations for digital health technology in older adult care: equity, privacy, and data responsibility. We also provide suggestions for future research and actions to address each ethical consideration before health technology should be broadly deployed in clinical contexts.

Technology equity requires equalising access to all elements required for technology use across people with varying technology literacy. These elements include high-quality internet; technology set-up and support; open-source data and algorithms; and program usability, adoption, and adaptability. Inequities are present in all of these domains. For example, older adults with lower income continue to have lower access to smartphones, tablets, computers, and home broadband.^8,9^

Researchers can integrate equity frameworks into health technology studies,^10^ and ensure that participant demographics, as well as digital literacy, are reported to help identify equity gaps.^11^ As prediction algorithms become more ubiquitous, there is an increased need to test these algorithms in participants with diverse backgrounds. Ensuring participants with diverse backgrounds are included in this type of research helps to mitigate the effects of algorithm biases and to ensure the lived experiences of these people are represented. Additionally, researchers must include members of key groups (eg, patients, caregivers, and clinicians) in the development of digital health technology for older adults, and must apply user-centred design strategies to ensure data are clinically useful and meaningful, especially for those most likely to struggle with technology.^3^ For instance, a 2021 report showed 39–45% of adults aged 50 years and older in the USA feel that technology today is not being designed with people of all ages in mind.^2^ Expanding participant inclusivity in technology research and design, particularly participants from systematically marginalised groups (eg, Black Americans), can allow for better representation of the range of digital literacy, technology access, and interest in digital health technology. Barriers in participant access to and utilisation of health technologies should be proactively identified and addressed before roll-out.

Upholding the highest standard of data privacy throughout the data lifecycle is crucial for health technologies. There are many data privacy concerns surrounding data ownership, transfer, and storage. Research that examines older adults’ perspectives on data privacy and their potential hesitations in using health technology due to data privacy concerns is needed, especially as continuous monitoring becomes integrated into the health-care system. Additionally, pre-emptive, transparent, and universal data privacy and security policies for all health technology, that encompass data collection, storage, transfer, raw data ownership, and sharing, are needed. At a minimum, all digital health technology (in the USA) that collects health data should meet Health Insurance Portability and Accountability Act standards for both research and non-research (eg, for-profit) applications. Transparent open-source algorithms are also needed for consistent detection and monitoring of health risks across health-care systems.

Health technologies will introduce substantial new data, along with a responsibility to respond to abnormal data, such as abnormally high heart rates or a fall. There is a risk of data overload for both patients and providers. Currently, there is no clinical infrastructure for integrating data into routine care, no guidance on clinician responsibility for monitoring remote data, few reimbursement policies for reviewing continuous data, and no clinician training for data review. For effective integration of digital health technology, health-care systems would need: protocols for responding to monitoring alarms; technology support systems for data extraction and protection, software upgrades, and user training; dashboards for integrating new data into electronic medical records; and mechanisms for reviewing and billing for data outside of health-care visits.

Implementation science studies can help determine how, when, and where data should fit into clinical workflows. Specifically, development of and research on robust data dashboards can help ensure efficiency and clinical utility. Additionally, it is necessary to evaluate the clinical value of data monitoring and subsequent responses to abnormal data before implementing technology into clinical practice. More economic studies outlining technology costs and benefits to the health-care system in the USA are needed.

Research targets to address the ethical issues discussed in this Comment are summarised in the figure.

## Figures and Tables

**Figure:**
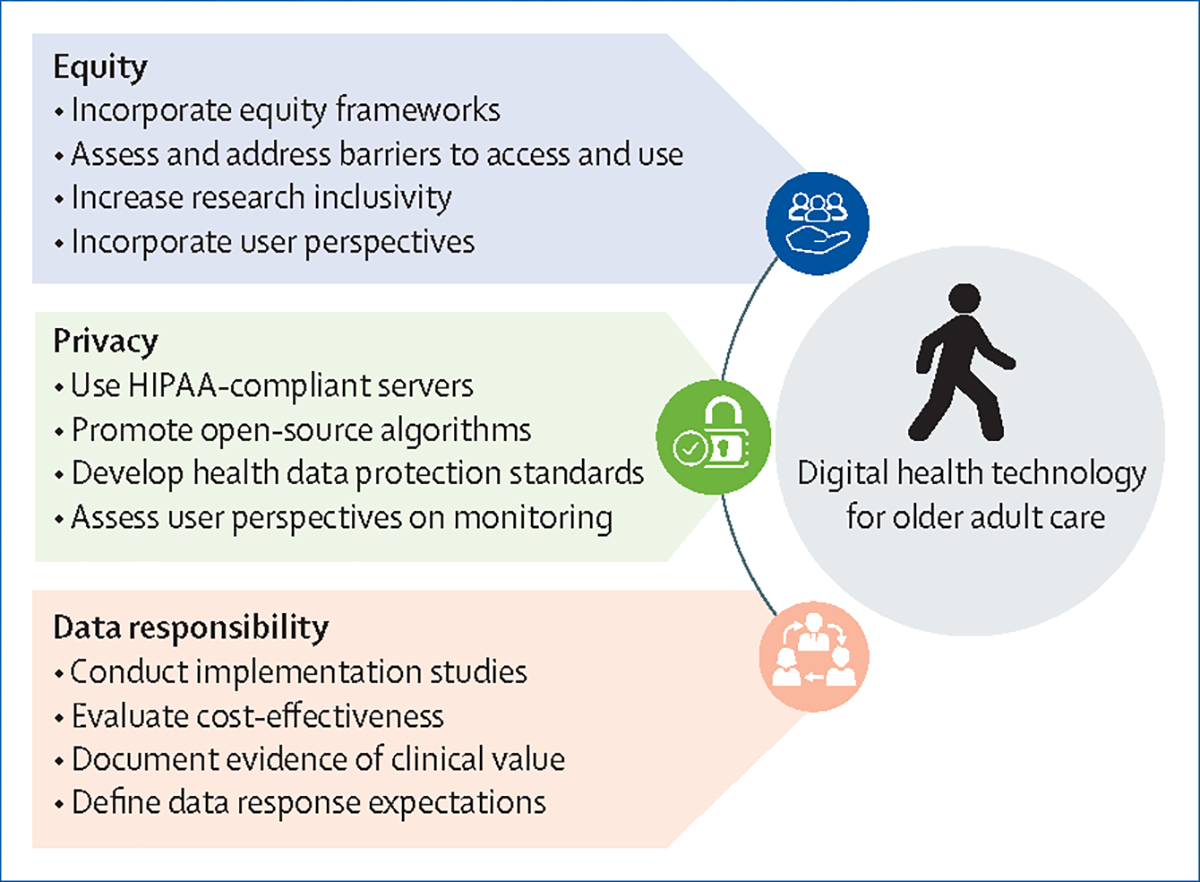
Research targets to promote ethical considerations of digital health technology in older adult care HIPAA=Health Insurance Portability and Accountability Act.
